# The Role of Music-Based Interventions in Orthopaedic Surgery

**DOI:** 10.7759/cureus.31157

**Published:** 2022-11-06

**Authors:** Benjamin Chiang, Caillin Marquardt, Jeffery C Martin, Alisa Malyavko, Sean Tabaie

**Affiliations:** 1 General Surgery, Riverside University Health System Medical Center, Riverside, USA; 2 Orthopaedic Surgery, George Washington University School of Medicine and Health Sciences, Washington DC, USA; 3 Orthopaedic Surgery, East Tennessee State University-Quillen College of Medicine, Johnson City, USA; 4 Orthopaedic Surgery, Children's National Hospital, Washington DC, USA

**Keywords:** patient-centred care, orthopaedic surgery, wellness resources, alternative therapies, music intervention

## Abstract

Music-based interventions (MBIs), such as music therapy, are interventions in which music is used to address the physical, emotional, and social needs of individuals. The origin of music-based therapy can be traced to ancient Egypt and expanded into the United States during the mid-1900s. These interventions have shown efficacy in reducing anxiety and pain in both nonsurgical and surgical settings across various medical specialities, one of which is orthopaedic surgery. Prior studies have investigated the use of MBI in adult and paediatric patients and have shown an improvement in patient well-being following medical care and a reduction in healthcare costs across both patient populations. This standard review covers the current utility of MBI in the field of orthopaedic surgery and explores the current literature on the application and limitations of MBI in both the operative and nonoperative aspects of orthopaedic care.

## Introduction and background

Music-based interventions (MBIs), such as music therapy (MT), are forms of therapy in which music is used to address the physical, emotional, cognitive, and social needs of individuals [1]. The idea of using music as a therapeutic agent in medicine stems from the influence of cultural, societal, and spiritual values on our understanding of how human minds detect and interpret music [2]. Early forms of MBI date back to ancient Egypt [2], and MBIs have been used in the United States since the 1950s [3]. Since then, music has found its way into rehabilitative medicine, orthopaedic surgery practices, psychological applications, and pain control [3]. The positive impact of MBI on the quality of life, anxiety, and social functioning of adult patients with chronic conditions has previously been demonstrated [4-6]. Specifically, a notable level of improvement in memory, orientation, depression, and anxiety was seen among patients with Alzheimer's disease who underwent at least four sessions of music therapy [4]. Additionally, some benefit in improving the quality of life for patients with schizophrenia was also found; however, the quality of evidence in the study was considered moderate to low [5]. MBI has also been shown to be effective in settings surrounding both nonsurgical and surgical procedures [7-8]. A meta-analysis investigating the impact of the musical intervention on postoperative pain following orthopaedic surgery found that there was a significant improvement in postoperative pain and anxiety following the procedures [7]. In children and adolescents, MBIs are both feasible and cost-effective interventions, particularly in mental health contexts, but also in procedural and surgical settings [9-14]. Our standard review of MBI in orthopaedic surgery aims to further elucidate the benefits and application of MBI in orthopaedic surgery for both adult and paediatric patients, further subdivided by orthopaedic subspecialty. We hypothesize that MBI has been shown to have similar positive impacts on both adult and paediatric orthopaedic patients as shown in previous studies for different specialities.

## Review

MBI in adult orthopaedics

MBIs have been studied in several adult orthopaedic subspecialties, as well as in the clinical and perioperative settings surrounding orthopaedic surgery [7,15]. The findings of these studies can be separated into three broad categories: whether MBI was helpful for psychological outcome measures (e.g. anxiety), physiologic measures (e.g. heart rate), or pain (e.g. visual analogue scale (VAS) (Table 1). Several different methods of assessment, such as the VAS, were used to assess various categories in prior studies. The notable results are depicted in Table 1 and have been categorized based on the intention of the original article.

**Table 1:** Summary table of included orthopaedic articles regarding music-based interventions, sorted alphabetically ^1^Not all outcome methods from every study are reported. ^2^Article is not primarily orthopaedic literature, although it had findings that can inform about the application of MBI in orthopaedics. AHTPS: Alder-Hey Triage Pain Score; NS: nonsignificant; NRS: numeric rating scale; MMSE: Mini-Mental State Exam; OSBD-r: Observational Scale of Behavioral Distress-Revised; STAI: State-Trait Anxiety Inventory; RCT: randomized controlled trial; VAS: visual analogue scale; TKA: total knee arthroplasty; postop: postoperative.

Author, year, journal	Orthopaedic subspecialty	Type of study, n, procedure, arms	Selected psychological outcome data	Selected physiological outcome data	Selected pain outcome data	Other outcomes
Main methods of assessment (p value)^1^						
Allred et al., 2010, Pain Manag Nurs [16]	Adult reconstruction	RCT, n=56 patients aged 46-84 years, receiving total knee arthroplasties, 2 arms: music listening sessions before and after the first ambulation vs control (20-minute quiet rest period)	VAS for anxiety (0.206)	Mean arterial pressure (0.000), heart rate (NS), respiratory rate (NS), oxygen saturation (NS)	VAS for pain (0.337), McGill pain questionnaire (not provided)	Administration of opioids (0.388)
Çetinkaya, 2019, J Perianesth Nurs [17]	Adult reconstruction	RCT, n=60 patients aged 65 years or older, undergoing total hip arthroplasty or total hip arthroplasty, 2 arms: music for three postoperative days vs control (no music)	Did not assess	Did not assess	Did not assess	MMSE (0.069) Neelon and Champagne Confusion Scale (0.000)
Ferraz et al., 2021, Eur J Pain [18]	Trauma	RCT, n=70 patients aged 18-60 years, with open diaphyseal tibial fracture undergoing wound care, 2 arms: music listening and standard analgesia vs control (standard analgesia only)	Did not assess	Did not assess	Pain NRS (<0.001)	Did not assess
Gallagher et al., 2018, Orthop Nurs [19]	Adult reconstruction shoulder/elbow	RCT, n=163 patients over 18 years of age, various joint replacement surgeries, 2 arms: visit by music therapist vs control (regular orthopaedic therapy)	Anxiety NRS (0.005), Rogers Faces Assessment Tool (<0. 001)	Nausea NRS (0.99)	Pain NRS (<0.001)	Did not assess
Kukreja et al., 2020, Cureus [20]	Adult reconstruction	RCT, n=57 patients over 18 years of age, undergoing total knee arthroplasty under spinal anaesthesia, 2 arms: music during TKA vs control (no music)	STAI State Postoperative (0.094), STAI Trait Postoperative (0.011)	Did not assess	Did not assess	Propofol dose (0.264), patient satisfaction (0.009)
Kwon et al., 2006, Taehan Kanho Hakhoe Chi [21]	Trauma	Quasi-experimental, n=60 patients aged 18-60 years, with surgery for leg fracture more than two weeks prior to therapy, 2 arms: music listening sessions vs control (standard care)	Levels of depression (0.558)	Systolic blood pressure (0.003), diastolic blood pressure (0.001), HR (0.000), respiration rate (0.000)	Pain questionnaire (0.000)	Discomfort level within- subjects over time (0.000)
Laframboise-Otto et al., 2021, Pain Manag Nurs [22]	Adult reconstruction	RCT, n=47 patients aged 18-90 years, undergoing hip or knee arthroplasty, 2 arms: music listening sessions postoperatively vs control (standard care)	Did not assess	Did not assess	Pain NRS at different time points: day of surgery (0.02), postop morning (0.04), postop noon (0.01), postop evening (0.21), postop day 2 (0.08, 0.31, 0.18), discharge day 1 and day 2 (significant at all time points)	Opioid analgesics (NS), nonopioid analgesics (NS)
Lin et al., 2011, J Clin Nurs [23]	Spine	Quasi-experimental, n=60 mean age 62.2 years, undergoing spine surgery; 2 arms: music listening sessions vs control (quiet environment)	STAI (0.074 to 0.286 at different time points), VAS for anxiety (<0.001 to 0.018 at different time points)	Heart rate (NS), systolic blood pressure (0.007 at 1 time point), diastolic blood pressure (NS), mean blood pressure (0.014 at 1 time point), 24-hour cortisol (NS), norepinephrine (NS), epinephrine (NS)	VAS for pain (<0.001 at all time points)	Did not assess
Liu et al., 2007, J Pediatr Orthop [24]	Paediatrics	RCT, n=69 aged 0.5 to 10 years, undergoing cast room procedures, 2 arms: soft lullaby music played in the background during procedure vs control (no music)	Did not assess	Heart rate in the waiting room (0.001), HR during the procedure (0.05)	Did not assess	Did not assess
McCaffrey and Locsin, 2004, J Clin Nurs [25]	Adult reconstruction	RCT, n=66 aged 65 years and over, undergoing elective hip or knee surgery, 2 arms: access to music on a CD vs control (no CD player)	Did not assess	Did not assess	Did not assess	Number of episodes of delirium and confusion (0.001) Readiness to ambulate (0.001)
Schneider, 2018, J Holist Nurs [26]	General	Quasi-experimental, n=42 patients with 65 pain score logs, aged 39-84 years with various orthopaedic surgeries, 2 arms: pain before music listening vs pain after music listening in the same patient	Did not assess	Did not assess	Pain NRS (before vs after MBI) (<0.001)	Did not assess
Sunitha Suresh et al., 2015, Pediatr Surg Int [27]	Pediatrics^2^	RCT, n=56 aged 6-18 years, undergoing various major surgical procedures, 3 arms: audiotherapy given post-procedure vs music therapy given post-procedure vs control (no intervention)	Did not assess	Did not assess	Pain burden between music and control groups (0.055)	Did not assess
Tolunay et al., 2018, Injury [28]	General	RCT, n=199 patients over 18 years of age, cast room procedures (intra-articular injection, fracture reduction, and cast removal), 2 arms: music during procedure vs control (no music)	STAI (0.032)	P-wave dispersion (0.225), corrected QT interval dispersion (0.031)	VAS (0.005)	Patient satisfaction (<0. 001), willingness of the patient to repeat procedure (<0. 001)
Townsend et al., 2021, J Hand Surg Glob Online [29]	Hand	RCT, n=50 mean age=60.8 years, undergoing wide-awake local anaesthesia no-tourniquet hand surgery; 2 arms: music and noise-cancelling headphones intraoperatively vs control (no music or headphones)	Intraoperative VAS for anxiety (0.017), postoperative VAS for anxiety (0.270)	Intraoperative systolic blood pressure change (0.422), intraoperative heart rate change (0.944)	Did not assess	Postoperative VAS for satisfaction (0.102)
van der Heijden et al., 2019, J Pediatr Psychol [30]	Pediatrics^2^	RCT, n=191 median age=7.3 years, undergoing various emergency room procedures, 3 arms: music vs cartoon watching vs control (no intervention)	OSBD-r during the procedure (0.55)	Heart rate before and after the procedure (0.450, 0.825)	AHTPS overall (0.003), facial pain scale-revised (0.077), AHTPS during plaster of Paris placement (0.004)	Did not assess
Wang and Tian, 2021, Comput Math Methods Med [31]	General	Quasi-experimental, n=38 patients, with 65 logs, 2 arms: subjects before listening to music vs patients after listening to music	Did not assess	Did not assess	Pain NRS (before vs after MBI) (<0.005)	Did not assess

MBI in the nonsurgical orthopaedic setting

MBI has the potential to be effective in the clinical setting in both the paediatric and adult orthopaedic populations. Tolunay et al. conducted a randomized controlled trial (RCT) of 199 adult patients during cast room procedures and found that patients had lower anxiety scores and VAS pain scores with a higher patient satisfaction [28]. In paediatric patients, Lin et al. conducted a similar RCT which assessed children undergoing cast room procedures. This study noted a decrease in heart rate in the pre-procedural setting and the procedural setting, which acted as a surrogate marker for anxiety in the study [7]. Additionally, children undergoing orthopaedic procedures in the emergency setting, such as cast application, have had significantly less pain in the setting of MBI. MBI was found to be better than other forms of distraction, such as watching cartoons, for minimizing pain in this setting [30]. Although the investigation of MBI in the nonsurgical orthopaedic setting is in its early phases, based on the findings in the surgical setting, there could be a great benefit of MBI in addressing in-office preprocedural and intraprocedural anxiety.

MBI in the surgical orthopaedic setting

In the orthopaedic surgical setting, MBI has been administered with different timing and frequency. Most commonly, MBI is administered during the recovery phase following surgery [7,16-19,21-22,25-27,31]. MBI has also been administered intraoperatively for patients undergoing surgeries either wide-awake under local anaesthesia or with spinal anaesthesia and has been associated with increased patient satisfaction scores (p=0.009) and reduced anxiety (p=0.017) post-operatively [20,29]. The methods of MBI vary widely as well, ranging from patients listening to music on their own [16-18,20-23,25-27,31], music being played ambiently [20], or official clinical visits by music therapists [19]. The genre of music administered in these sessions is also diverse: from popular music to traditional folk music [20,23]. Finally, MBI has been administered for patients undergoing a variety of procedures across orthopaedic subspecialties including adult reconstruction [16-19,21-25], foot and ankle [26-27,31], hand [29], shoulder and elbow [19], spine [23], and trauma [18,21,31].

As mentioned above, MBI has also recently been studied in the context of improving the patient experience for orthopaedic surgeries performed when the patient is awake. Anecdotally, patients will sometimes request music for these types of procedures which is usually accomplished by having a nearby mobile device or headphones. A study done by Kukreja et al. investigated total knee arthroplasty patients with spinal anaesthesia and found that the postoperative State-Trait Anxiety Inventory (STAI) Trait score was significantly reduced when music was played during the surgery (p=0.011) [20]. A similar 2021 study by Townsend et al. on the efficacy of MBI via headphones during wide-awake local anaesthetic no-tourniquet hand surgeries demonstrated lowered intraoperative anxiety [29]. These studies lay a foundation for the applicability of MBI intraoperatively and contribute to the already existing literature about intraprocedural MBI. This application of MBI suggests that MBI may be helpful for anxiety and pain in many more untested settings. For example, MBIs during patient transfers from the pre-procedure unit to the operating theatre or from the operating theatre to the post-anaesthesia care unit have not yet been studied.

MBI and its effect on pain and anxiety in the orthopaedic setting

MBI, in its various forms of administration, was helpful for lowering subjective pain scores in the postoperative period [18-19,21-22,26,31]. The impact of MBI is most often recorded using a patient-assessed numeric rating scale (NRS) [18-19,22,26,31] in addition to the VAS, which has been utilized as a measure in several studies [16,23]. Notably, all studies which used NRS found significant reductions in pain with MBI [18-19,21-22,25-27,31]. Meanwhile, the studies which used VAS as the measure for pain did not find a significant reduction in pain with MBI [16,23]. Postoperative pain questionnaires have also been used to assess this outcome [21]. Laframboise-Otto et al. [22] aimed to establish a timeline for the efficacy of MBI and provide evidence that it is efficacious for pain relief for up to the first postoperative day during orthopaedic admissions as well as after discharge. However, this study was limited to postoperative evaluation of only patients who underwent total joint arthroplasty [22]. A similar trend has been found regarding the reduction of negative psychological outcomes in the postoperative period. Psychological outcomes were assessed using anxiety NRSs [19], the State-Trait Anxiety Inventory (STAI) [20,23,29], and VAS for anxiety [16,23,29,32] (Table 1). Various incongruities exist in the measures of pain and anxiety as well as the method of administration of MBI in these studies. One study measuring the level of postoperative depression did not find a significant difference in the improvement of depression when comparing the control cohort to patient scores in the MBI cohort. However, this study did show that the score attained by patients in the MBI cohort on the depression scale had a greater reduction than those in the control cohort [21].

Taking into account all prior studies investigating MBI, it seems likely that MBI is helpful for lowering the subjective postoperative pain and anxiety for patients. With the ever-growing importance of patient-reported outcome measures in orthopaedic surgery [32], MBI, which is both feasible and cost-effective, should be considered by orthopaedic departments in the immediate postoperative recovery period.

MBI for other purposes in orthopaedics

Studies have also characterized the utility of MBI for other outcomes following orthopaedic surgery, such as delirium and confusion [17,25], opioid use [16,20,22], and patient satisfaction [16,20,29]. An early study by McCaffrey and Locsin on delirium and confusion showed a decrease in episodes of delirium and confusion in patients with MBI following orthopaedic surgery (p=0.001) [25]. Another study by Cetinkaya [17] performed in 2019 investigated whether three days postoperatively MBI would be able to reduce delirium. They determined that patients with MBI were less confused based on the Neelon and Champagne Confusion Scale (NEECHAM) (p=0.001) [17]. Unfortunately, MBI has not been shown to reduce opioid requirements in any of the included studies [16,20,22]. The investigation into whether MBI can lower opioid requirements is an attempt to provide a more objective measure for pain. Perhaps RCTs or database studies investigating opioid use following orthopaedic surgery can begin to elucidate the benefit of MBI for this measure. Finally, a higher patient satisfaction for orthopaedic procedures has been associated with MBI based on patient questionnaires [16,20], but not when assessed with VAS [29].

The future of MBI in orthopaedics

Since many studies have found MBI to be helpful in recovery following orthopaedic surgery, future directions for the investigation of MBI should focus on whether there are differences depending on the form of administration, the type of music, and the frequency of music exposure (preoperative, intraoperative, and postoperative settings). Given the diversity of studies already available about the general applicability of MBI, MBI in the current orthopaedic setting can be tailored to patient preferences at this time as future research proceeds.

Bias of reviewed studies

Many of the included studies had difficulty with the elimination of bias (Table 2) [33]. Blinding outcome assessors was the most difficult, with most of the included studies showing a high risk of bias in this category [16-22,28,30]. As previously mentioned, NRS and STAI were common measures of pain and anxiety in MBI studies. Unfortunately, both of these measures require patients to be the primary outcome assessors. Since patients cannot be blinded to whether or not they received MBI, any study that used patient-guided assessments as part of their methodology had this risk of bias. This is particularly evident in the outcomes for pain, in which studies that utilized NRS were statistically significant, but not in studies that utilized VAS scores. Future studies should consider only using professionally administered rating scales for study outcomes, such as the VAS, or objective measures of pain measurement such as a quantitative measure of opioids used. At the same time, the reality is that pain is subjective, and although methods such as opioid use can be used to quantify pain, studies that allow patients to grade their own pain might be a closer reflection of how patients truly assess their own wellbeing.

**Table 2:** Risk bias assessment of included articles, sorted alphabetically

Author, year, journal	Random sequence generation	Allocation concealment	Performance bias	Blinding outcome assessors	Incomplete outcome data (attrition bias)	Selective reporting
Allred et al., 2010, Pain Manag Nurs [16]	Low	Low	Low	High	High	High
Çetinkaya, 2019, J Perianesth Nurs [17]	Low	Low	High	High	Low	Low
Ferraz et al., 2021, Eur J Pain [18]	Low	Low	Low	High	Low	Low
Gallagher et al., 2018, Orthop Nurs [19]	Low	Low	High	High	Low	Low
Kukreja et al., 2020, Cureus [20]	Low	Low	High	High	High	High
Kwon, 2006, Taehan Kanho Hakhoe Chi [21]	High	Unclear	High	High	Low	High
Laframboise-Otto et al., 2021, Pain Manag Nurs [22]	Low	Low	High	High	Low	High
Lin, 2011, J Clin Nurs [23]	High	Unclear	Low	High	Low	Low
Liu, 2007, J Pediatr Orthop [24]	High	High	High	High	Low	Unclear
McCaffrey and Locsin, 2004, J Clin Nurs [25]	Unclear	Low	Low	Low	Low	Low
Schneider, 2018, J Holist Nurs [26]	High	Unclear	Low	High	Low	Low
Sunitha Suresh et al., 2015, Pediatr Surg Int [27]	Unclear	High	High	Low	Low	Low
Tolunay et al., 2018, Injury [28]	Low	Low	Low	High	High	Low
Townsend, 2021, J Hand Surg Glob Online [29]	Unclear	Low	High	Low	Low	Low
van der Heijden et al., 2019, J Pediatr Psychol [30]	Low	Low	High	High	Low	Low
Wang and Tian, 2021, Comput Math Methods Med [31]	High	High	High	High	High	Low

Performance bias was also high in several studies and particularly difficult to eliminate in studies where it was obvious that the patient was receiving MBI (e.g. ambient music, headphones playing, official visits from a music therapist) [17,19-22,24,27,30]. Specifically, studies evaluating MBI in the intraoperative setting and intraprocedural setting have difficulty eliminating this form of bias [20,24,29]. This again highlights the difficulty of assessing and studying different forms of administration of MBI as study designers may tend to favour self-administered MBI, which is easier to blind for performance bias, while other forms of MBI that are harder to administer or blind for may in actuality be more helpful to patients.

## Conclusions

In conclusion, MBIs have been used in many orthopaedic settings: clinical procedures, preoperatively, intraoperatively, and postoperatively. Prior studies have found that different forms of MBI do have notable effects on reducing postoperative pain and anxiety in surgical settings. MBI has been found to be beneficial in both the adult and paediatric population which can help contribute to reducing additional medical interventions. By reducing additional medical intervention, such as the need for long-term pain management, MBI can help improve patient satisfaction and reduce overall healthcare costs to both the patient and the healthcare system. However, the diversity of MBI studies in current literature makes it somewhat difficult to directly compare outcomes, and many studies have had difficulty reducing bias due to the nature of MBI. More research is necessary to better ascertain the benefits of MBI and to fine-tune the application of MBI in various orthopaedic settings. At this time, MBI should be considered on a patient-by-patient basis in orthopaedic surgery to reduce patient pain and anxiety while improving patient satisfaction with their medical care.
